# Altered cerebral blood flow in patients with spinocerebellar degeneration

**DOI:** 10.3389/fnins.2022.977145

**Published:** 2022-09-13

**Authors:** Bing Liu, Aocai Yang, Wenwen Gao, Yue Chen, Yige Wang, Xiuxiu Liu, Kuan Lv, Linwei Zhang, Guolin Ma

**Affiliations:** ^1^Department of Radiology, China-Japan Friendship Hospital, Beijing, China; ^2^Graduate School of Peking Union Medical College, Chinese Academy of Medical Sciences and Peking Union Medical College, Beijing, China; ^3^Department of Radiology, Peking University China-Japan Friendship School of Clinical Medicine, Beijing, China; ^4^Department of Neurology, China-Japan Friendship Hospital, Beijing, China

**Keywords:** spinocerebellar degeneration, spinocerebellar ataxia, cerebral blood flow, arterial spin labeling, cerebellum

## Abstract

**Objectives:**

Spinocerebellar degeneration (SCD) comprises a multitude of disorders with sporadic and hereditary forms, including spinocerebellar ataxia (SCA). Except for progressive cerebellar ataxia and structural atrophy, hemodynamic changes have also been observed in SCD. This study aimed to explore the whole-brain patterns of altered cerebral blood flow (CBF) and its correlations with disease severity and psychological abnormalities in SCD *via* arterial spin labeling (ASL).

**Methods:**

Thirty SCD patients and 30 age- and sex-matched healthy controls (HC) were prospectively recruited and underwent ASL examination on a 3.0T MR scanner. The Scale for Assessment and Rating of Ataxia (SARA) and the International Cooperative Ataxia Rating Scale (ICARS) scores were used to evaluate the disease severity in SCD patients. Additionally, the status of anxiety, depression and sleep among all patients were, respectively, evaluated by the Self-Rating Anxiety Scale (SAS), Self-Rating Depression Scale (SDS) and Self-Rating Scale of Sleep (SRSS). We compared the whole-brain CBF value between SCD group and HC group at the voxel level. Then, the correlation analyses between CBF and disease severity, and psychological abnormalities were performed on SCD group.

**Results:**

Compared with HC, SCD patients demonstrated decreased CBF value in two clusters (FWE corrected *P* < 0.05), covering bilateral dentate and fastigial nuclei, bilateral cerebellar lobules I-IV, V and IX, left lobule VI, right lobule VIIIb, lobules IX and X of the vermis in the cerebellar Cluster 1 and the dorsal part of raphe nucleus in the midbrain Cluster 2. The CBF of cerebellar Cluster 1 was negatively correlated with SARA scores (Spearman’s rho = –0.374, *P* = 0.042) and SDS standard scores (Spearman’s rho = –0.388, *P* = 0.034), respectively. And, the CBF of midbrain Cluster 2 also had negative correlations with SARA scores (Spearman’s rho = –0.370, *P* = 0.044) and ICARS scores (Pearson *r* = –0.464, *P* = 0.010).

**Conclusion:**

The SCD-related whole-brain CBF changes mainly involved in the cerebellum and the midbrain of brainstem, which are partially overlapped with the related function cerebellar areas of hand, foot and tongue movement. Decreased CBF was related to disease severity and depression status in SCD. Therefore, CBF may be a promising neuroimaging biomarker to reflect the severity of SCD and suggest mental changes.

## Introduction

Spinocerebellar degeneration (SCD) comprises a group of rare neurodegenerative diseases, including sporadic SCD [e.g., multiple system atrophy (MSA) cerebellar type] and hereditary SCD (e.g., autosomal dominant or recessive cerebellar ataxias). Neuropathological studies in SCD have revealed neuronal cell loss in the cerebellum, brainstem and spinal cord, occasionally in the basal ganglia and cerebral cortex (Yamada et al., 2016; Iwabuchi et al., 2022). Generally the cerebellum is most severely involved, with progressive cerebellar ataxia and atrophy as the main characteristics of SCD, but patients may also appear truncal and limb ataxias, dysarthria, dysphagia, pyramidal and extrapyramidal signs, oculomotor deficits (nystagmus, hypermetria/hypometria of saccades), and autonomic disorders (Kawai et al., 2009). In addition, patients with SCD may have a higher prevalence of non-motor psychological symptoms such as depression, anxiety and sleep disorders which lead to the strong negative impact on their quality of life (Pedroso et al., 2011; Silva et al., 2015; Mastammanavar et al., 2020). Therefore, particular attention should also be paid to the mental health of SCD patients. Except for brain structural alterations affected by local pathology, these clinical manifestations and psychological impairments are likely to be associated with brain functional changes as well because the brain activity measured by hemodynamics or metabolism can reflect the underlying neuronal events. One of the important indicators for hemodynamic change reflection and cerebral perfusion evaluation is the cerebral blood flow (CBF) value. Cerebellar hemodynamic changes such as hypoperfusion have been detected in SCD patients with decreased CBF measured by positron emission tomography (PET) or single-photon emission computed tomography (SPECT) (Kondo et al., 1993; Gilman et al., 1995; De Michele et al., 1998; Honjo et al., 2004), indicating the reduction in neuronal activity. Although PET has been regarded as the gold standard for CBF measurement, ionizing radiation and contrast injection during PET/SPECT become the bottleneck of its extensive application in clinical routine.

Nowadays, as a completely non-invasive and non-radiative magnetic resonance imaging (MRI) technique with magnetically labeled blood water rather than exogenous contrast agents serving as the flow tracer, arterial spin labeling (ASL) imaging (Detre et al., 1992) has increasingly become the proxy measure for blood flow to provide MR-based CBF quantification. Currently, pseudo-continuous ASL (PCASL) (Dai et al., 2008) is the first choice for clinical application (Alsop et al., 2015) due to its higher signal-to-noise ratio (SNR). Demonstrating good correlation and agreement with PET and SPECT (Detre and Alsop, 1999; Ikawa et al., 2018), ASL MRI has already been used for evaluating the aberrant cerebral perfusion in neurodegenerative diseases such as Parkinson’s disease (PD) and MSA (Lin et al., 2017; Erro et al., 2020; Ivanidze et al., 2020), but rarely in SCD. A Previous study retrospectively estimated CBF values in 4 cerebellar regions of 16 patients with SCD and demonstrated lower cerebellar CBF (Ikawa et al., 2018). Another study (Xing et al., 2014) had applied ASL on one specific subtype of SCD, namely spinocerebellar ataxia (SCA) type 3 (SCA3), and found decreased regional CBF (rCBF) in the pons, cerebellar dentate nucleus and cerebellar cortex of the onset SCA3 group. However, these studies only analyzed CBF values that were extracted from the predefined regions of interest (ROIs), without considering the whole brain. Given that SCD contains various subtypes and each subtype might have its own representative brain regions, the selection of ROIs becomes difficult in patients with SCD and the results of perfusion pattern would be influenced by the chosen ROIs. To our knowledge, the commonality of CBF alteration explored at the whole-brain level and the associations between hemodynamics and clinical measures (especially psychological scales) in SCD remain largely unknown.

As a data-driven method, voxel-based whole-brain analysis can investigate the spatial specificity of CBF changes without being limited to specific brain regions, which may be more appropriate in the comprehensive comparison between patients with SCD and the healthy controls. Besides, given the importance of visualization in neuroimaging, CBF results should be displayed appropriately, especially in the infratentorial space. To figure this out, our study applied a high-resolution spatially unbiased atlas template of the cerebellum and brainstem spatially unbiased infratentorial template (SUIT) (Diedrichsen, 2006) to better characterize cerebellar CBF alterations in SCD patients. Therefore, this study aimed to investigate the whole-brain pattern of altered CBF at the voxel level in patients with SCD by using the three-dimensional (3D) PCASL. Normalized CBF was used for narrowing the inter-subject difference. And we further explored the associations between CBF changes and clinical measures (especially the disease severity and psychological abnormalities) of SCD in brain regions with altered hemodynamics through regional analysis.

## Materials and methods

### Subjects

This study was approved by the Ethics Committee of China-Japan Friendship Hospital. All subjects had obtained written informed consent.

From June 2021 to November 2021, 30 SCD patients (20 males and 10 females; mean age, 45.6 ± 12.8 years; age range, 25–67 years; median disease duration, 6 years) and 30 age- and sex-matched healthy controls (HC) (18 males and 12 females; mean age, 45.6 ± 14.9 years; age range, 24–68 years) were prospectively recruited into this study at the China-Japan Friendship Hospital. Twenty six of the 30 SCD patients were diagnosed as hereditary SCD, including 23 SCA patients (SCA1, *n* = 1; SCA2, *n* = 2; and SCA3, *n* = 20) and 3 autosomal recessive cerebellar ataxias (ARCA) patients (*ANO10*-ARCA, *n* = 1; *SPG7*-ARCA, *n* = 1; *NPC1*-ARCA, *n* = 1). And the other 4 patients were diagnosed with sporadic MSA cerebellar type (MSA-C). The inclusion criteria for patients were: (1) diagnosed as SCD by trained neurologists according to the neurological manifestations, genetic tests, family history, and routine brain MRI findings, thereinto, the hereditary SCD and its specific subtypes (SCA or ARCA) should be proven by genetic testing and the sporadic MSA-C (probable or possible) should be diagnosed by the second consensus statement of 2008 (Gilman et al., 2008); (2) age ≥ 18 years; (3) right-handed. The exclusion criteria for patients were as follows: (1) with contraindications to MRI examination; (2) having the history of other neurological diseases, such as brain trauma, strokes and tumors; (3) artifacts in MRI images. Recruitment of HC from the local communities was based on the following inclusion criteria: (1) without the history of any neuropsychiatric disorders; (2) age ≥ 18 years; (3) right-handed. The exclusion criteria for HC were the same as that for SCD patients.

### Clinical characteristics

Demographic information collection and neurological evaluation were performed on the MRI scanning day, before the MR data acquisition. The Scale for Assessment and Rating of Ataxia (SARA) (Schmitz-Hübsch et al., 2006) and the International Cooperative Ataxia Rating Scale (ICARS) (Trouillas et al., 1997) scores were used to quantify neurological manifestations and reflect the disease severity in SCD patients. In addition, we evaluate the status of depression, anxiety and sleep among all patients according to the Self-Rating Anxiety Scale (SAS) (Zung, 1971), Self-Rating Depression Scale (SDS) (Zung, 1972) and Self-Rating Scale of Sleep (SRSS) (Li, 2012) to reflect psychological impairments in SCD group. For SAS and SDS, we used the standard score rather than the total score for anxiety and depression status assessment. Only when the standard SAS score ≥ 50 or the standard SDS score ≥ 53 could patients themselves be considered in anxious or depressed status, respectively.

### Magnetic resonance imaging data acquisition

MRI scans of all subjects were performed on a 3.0T MR scanner (General Electric, Discovery MR750, Milwaukee, WI, United States) with an 8-channel head coil. Acquisition parameters for resting-state 3D PCASL were: slice thickness = 4 mm, repetition time (TR) = 4,817 ms, echo time (TE) = 14.6 ms, flip angle = 111°, post label delay = 1,525 ms, spiral in readout of 12 arms with 1,024 sample points, field of view (FOV) = 240 mm × 240 mm, voxel size = 1.875 mm × 1.875 mm, number of excitations (NEX) = 3, matrix = 128 × 128. The total acquisition time for the resting state ASL scan was 6 min 54 s. Earplugs and paddings were provided to all subjects for noise reduction, and foam paddings were given to restrict head motion. During the resting-state ASL scans, all subjects were asked to keep their eyes closed without falling asleep, relax and think nothing special.

### Reconstruction and post processing of cerebral blood flow

Briefly, the PCASL difference images were calculated after the subtraction of the label images from the control images. The CBF maps were subsequently derived from the ASL difference images with Functool perfusion software in GE (General Electric Healthcare). The detailed calculation procedures have been described in a previous study (Xu et al., 2010).

CBF image processing and further voxel-based analysis were handled in a MATLAB-based software, Statistical Parametric Mapping 8 (SPM8).^1^ First, the CBF images of all subjects were non-linearly coregistered to a PET-perfusion template of SPM8 and spatially warped into the Montreal Neurological Institute (MNI) space, meanwhile the voxel size was resampled into 2 mm × 2 mm × 2 mm. After the spatial standardization, the CBF value of each voxel across the whole brain was normalized by the mean division method (Aslan and Lu, 2010). And a Gaussian kernel of 8 mm × 8 mm × 8 mm full-width at half maximum (FWHM) was applied for the spatial smooth to improve the whole-brain statistical analysis.

Each cluster with significant group differences compared in the voxelwise whole-brain manner would be regarded as the ROI in the following regional CBF analyses. A binary mask of the cluster ROI was firstly generated by the xjView toolbox (Version 10.0)^2^ and then loaded into Data Processing and Analysis of Brain Imaging (DPABI, Version 5.1) (Yan et al., 2016) toolbox to calculate the normalized CBF values within that mask. For each subject, the mean normalized CBF of each significant cluster was extracted and used for ROI-based regional analyses.

### Statistical analysis

#### Whole-brain analyses of cerebral blood flow

Voxel-based intergroup differences in normalized CBF values of whole-brain were analyzed using independent two-sample *t*-test in SPM8. Age and sex were induced as the nuisance variables in statistical model. Correction of multiple comparisons was executed using the family-wise error (FWE) method with a corrected voxel-level threshold of *P* < 0.05 and cluster size > 10 voxels.

#### Regional analyses of cerebral blood flow

ROI-based intergroup comparisons were analyzed by two-sample *t*-test in the SPSS software (Version 26.0, IBM Corp., Armonk, NY, United States). For SCD group, the ROI-based Pearson or Spearman correlation analyses were performed between normalized CBF values and clinical features (including disease duration, SARA scores, ICARS scores, SDS standard scores, SAS standard scores and SRSS scores). Significant threshold was set at *P* < 0.05.

#### Other statistical analyses

Other statistical analyses were also performed in the SPSS software, with graphs drawn in GraphPad Prism 9 (GraphPad Software, San Diego, CA, United States). The normality of data distribution was evaluated by the Shapiro-Wilk normality test. As for demographic comparisons between SCD patients and HCs, student’s *t*-test was used for age and Pearson Chi-square (χ2) test was used for sex. Significant threshold was set at *P* < 0.05.

### Visualization

Significant clusters of the whole-brain voxel-wise CBF analysis were visualized using MRIcron.^3^ And clusters that located at the cerebellum were overlaid onto the coronal SUIT (Diedrichsen et al., 2009) to better display the involvement of the cerebellar lobules as well as the deep cerebellar nuclei. Specifically, the cerebellar-located clusters were also projected to the surface-based cerebellar flatmap (Diedrichsen and Zotow, 2015) using SUIT toolbox (Version 3.4)^4^ implemented in SPM. The outline of surface-based clusters was further overlaid onto flatmaps for the anatomical orientation of cerebellar lobules I-X (both hemispheric and vermal) and the cerebellar movement orientation (Diedrichsen and Zotow, 2015), respectively.

## Results

### Demographic information and clinical characteristics

Demographic information and clinical characteristics of all SCD patients and healthy controls (HCs) are summarized in Table 1, with normal data shown in mean ± standard deviation (SD) and non-normal data shown in median (quartile). No significant group differences were found in age (*P* = 0.993) or sex (*P* = 0.592) between SCD group and HC group. In patients with SCD, disease duration ranged from 0 to 36 years with a median of 6 years. Total SARA scores of SCD group ranged between 1 and 36 with a median of 13, while total ICARS scores ranged from 0 to 88 with a mean of 41.0 (*SD* = 22.0). For three self-rating scales, the range of SAS standard scores was 25–71 with a mean of 47.8 (*SD* = 12.0); the range of SDS standard scores was also 25–71 with a median of 53, and that of SRSS scores was 12–43 with a mean of 25.7 (*SD* = 8.1). In addition, 15 of 30 SCD patients’ SAS standard scores were 50 or more. And the percentage of patients with SDS standard score ≥ 53 was 50% as well. We also collected data for the presence of additional neurological manifestations (e.g., truncal and limb ataxias: 96.7%, pyramidal and extrapyramidal signs: 46.7%, oculomotor deficits: 86.7%, dysarthria: 86.7%, dysphagia: 46.7%, and autonomic disorders: 13.3%).

**TABLE 1:** The demographical information and clinical characteristics of SCD group and HC group.

	SCD group (*n* = 30)	HC group (*n* = 30)	*p*-value
Age (years)	45.6 ± 12.8	45.6 ± 14.9	0.993^a^
Sex (M/F)	20/10	18/12	0.592^b^
Disease duration (years)	6.0 (3.0–10.0)^c^	N/A	N/A
SARA score	13.0 (10.0–20.5)^c^	N/A	N/A
ICARS score	41.0 ± 22.0	N/A	N/A
SAS standard score	47.8 ± 12.0	N/A	N/A
SDS standard score	53.0 (39.5–63.8)^c^	N/A	N/A
SRSS score	25.7 ± 8.1	N/A	N/A
Truncal and limb ataxias (*n*)	29 (96.7%)	N/A	N/A
Pyramidal and extrapyramidal signs (*n*)	14 (46.7%)	N/A	N/A
Oculomotor deficits (*n*)	26 (86.7%)	N/A	N/A
Dysarthria (*n*)	26 (86.7%)	N/A	N/A
Dysphagia (*n*)	14 (46.7%)	N/A	N/A
Autonomic disorders (*n*)	4 (13.3%)	N/A	N/A

^a^Two-sample student’s t-test; ^b^Pearson chi-squared test; ^c^Non-normal data is shown as Median (Q1–Q3). Q1, first quartile; Q3, third quartile. M, male; F, female; SCD, spinocerebellar degeneration; SARA, scale for assessment and rating of ataxia (0 = no ataxia, 40 = most severe ataxia); ICARS, international cooperative ataxia rating scale (0 = no ataxia, 100 = most severe ataxia); SAS, self-rating anxiety scale; SDS, self-rating depression scale; SRSS, self-rating scale of sleep; N/A, not applicable.

### Group differences in normalized cerebral blood flow

#### Whole-brain voxel-based analyses

In the whole-brain voxel-based analyses, the CBF group differences between the SCD patients and the HCs are shown in Table 2. For HC > SCD comparison, voxels that survived after the FWE correction at a voxel-level threshold of *P* < 0.05 and cluster size > 10 are mainly gathered in two clusters, namely Cluster 1 and Cluster 2. Cluster 1 was located at the cerebellum, with the vermis and bilateral cerebellar hemispheres involved, including AAL3 brain regions such as bilateral Cerebellum_4_5, Cerebellum_9 and Cerebellum_3, left Cerebellum_6, right Cerebellum_8, Vermis_4_5, Vermis_3, Vermis_10, Vermis_9 and Vermis_1_2. Other anatomy structures involved in Cluster 1 but not in AAL3 were culmen, dentate nuclei, cerebellar tonsil, fastigial nuclei, nodule, and declive. While Cluster 2 was found in the midbrain of brainstem with one meaningful AAL3-specific brain label Raphe_D, known as the dorsal part of raphe nucleus. For HC < SCD contrast, however, no brain regions or even voxels showed significantly increased CBF in SCD patients compared to HCs after FWE correction.

**TABLE 2:** Brain regions with voxel-based significant group differences in normalized CBF.

Contrast	Cluster	Total voxels	*t*-value	Height threshold *T* = 4.973 [voxel level, *P* < 0.05 (FWE), k = 0, cluster size > 10]		Peak MNI coordinate (mm)		
				Brain region	No. of significant voxels	x	y	z
HC > SCD	Cluster 1	1,329	6.920	Culmen	585	10	-46	-18
				Vermis_4_5*	173			
				Cerebellum_4_5_L*	160			
				Dentate nuclei	143			
				Cerebellum_4_5_R*	123			
				Cerebellum_9_R*	103			
				Cerebellar tonsil	81			
				Fastigial nuclei	75			
				Nodule	73			
				Vermis_3*	67			
				Declive	53			
				Cerebellum_9_L*	49			
				Vermis_10*	40			
				Cerebellum_3_R*	36			
				Cerebellum_3_L*	27			
				Vermis_9*	24			
				Vermis_1_2*	24			
				Cerebellum_6_L*	21			
				Cerebellum_8_R*	16			
HC > SCD	Cluster 2	95	6.192	Midbrain	67	2	-26	-4
				Raphe_D*	11			

*Brain labels defined in AAL3. The Arabic numerals in the AAL3 brain labels indicated the lobule number of the cerebellar hemisphere or vermis. For example, the anatomical description for Cerebellum_4_5_L was the lobule IV, V of the left cerebellar hemisphere.

**FIGURE 1:**
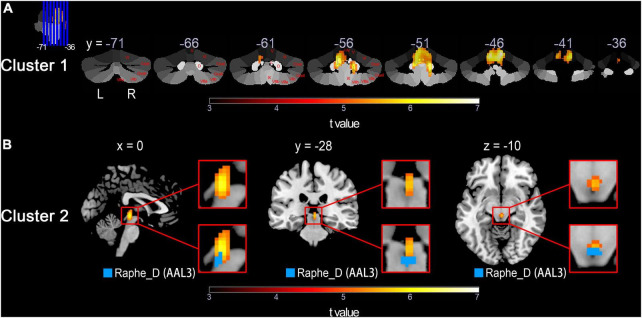
Detailed visualization for voxel-based HC > SCD comparison across groups using normalized CBF values performed by the two-sample *t*-test with a threshold of *P* < 0.05 (FWE corrected) and cluster size of 10 voxels. The warm color represents voxels with significantly decreased CBF in the SCD group compared with the HC group. Results of the HC < SCD comparison are not shown because no significant voxel was detected under the same FWE correction threshold and cluster size. **(A)** Cluster 1 shown in yellow/red, overlaid onto the cerebellar atlas (Diedrichsen et al., 2009) in coronal slices. Cerebellar lobules and nucleus are marked in red on the right cerebellum. **(B)** Top row: magnified Cluster 2 shown in yellow/red; Bottom row: Cluster 2 with AAL3 brain region mapped on. HC, healthy control; SCD, spinocerebellar degeneration; CBF, cerebral blood flow; FWE, family-wise error; Roman numerals I-X, number of cerebellar hemisphere lobules (Schmahmann et al., 1999); D, dentate nucleus; F, fastigial nucleus; Raphe_D, dorsal raphe nucleus (Rolls et al., 2020); L, left; R, right; x,y,z, space coordinates.

**FIGURE 2:**
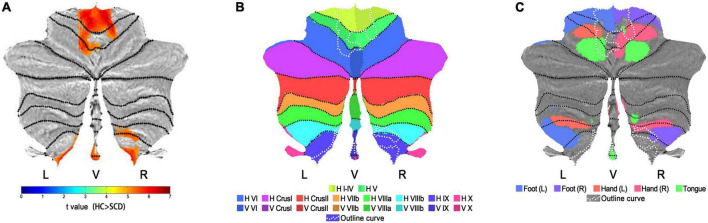
Visualization on flatmaps for cerebellar-located Cluster 1. **(A)** The t-map of Normalized CBF group results (HC > SCD) projected to the cerebellar SUIT flatmap. Regions displayed in warm colors represent the significant CBF decrease in SCD patients. **(B)** Outline curves of results in flatmap **(A)** shown in white dotted lines are overlaid onto the flatmap of anatomical distributions for cerebellar lobules I-X. Note that for lobule VI-X, two hemispheric and one vermal compartment (displayed in slightly different colors) are defined. **(C)** Outline curves of results in flatmap **(A)** shown in white dotted lines are overlaid onto the flatmap of the sensorimotor topography for activation related to hand, foot and tongue movement activations. HC, healthy control; SCD, spinocerebellar degeneration; L, left; R, right; V, vermis; H, hemisphere; Roman numerals I-X, number of cerebellar lobules (Schmahmann et al., 1999).

Figures 1, 2 show the detailed visualization for Cluster 1 and Cluster 2. In Figure 1A, the cerebellar-located Cluster 1, overlaid onto the SUIT template, shows its cover of bilateral dentate and fastigial nuclei, bilateral cerebellar lobules I-IV, V and IX, left lobule VI and right lobule VIIIb. While in Figure 1B, the smaller Cluster 2 is located on the midbrain of brainstem; and the AAL3 brain region Raphe_D is overlaid onto Cluster 2, showing its position in the midbrain. As the vermis has not been well demonstrated in the coronal slice view in Figure 1A, we further plotted the *t*-values of Cluster 1 to the SUIT flatmap, and generated a flat t-map displayed in Figure 2A. The outline of surface-based Cluster 1 is overlaid onto the anatomical orientation of cerebellar lobules I-X (both hemispheric and vermal) as well as the cerebellar movement orientation (Diedrichsen and Zotow, 2015) to form Figures 2B,C, respectively. According to Figure 2B, Cluster 1 also covers lobules IX and X of the vermis besides cerebellar hemispheric regions. And Figure 2C indicates the motor involvement of the foot (bilateral), hand (bilateral, more severe in right) and tongue.

#### Regional analyses

**FIGURE 3:**
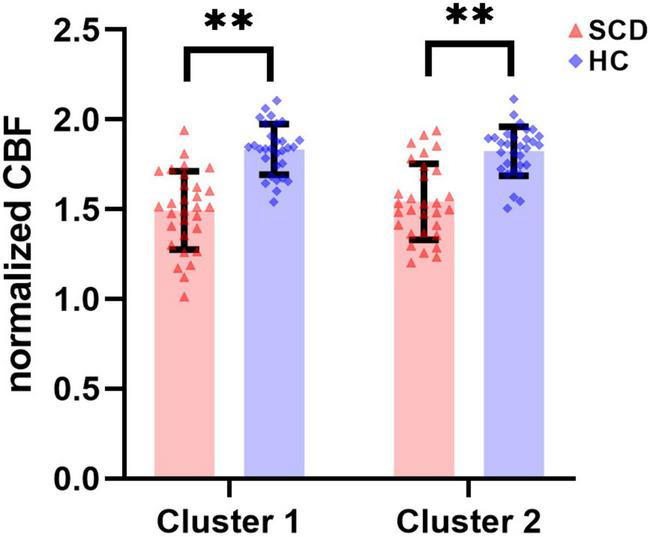
There were statistically significant differences in normalized CBF of ROI-based Cluster 1 and Cluster 2 between the SCD group and the HC group. SCD, spinocerebellar degeneration. ^**^ indicates *P* < 0.0001.

In the regional analyses based on the ROIs of significant clusters, results about intergroup comparison of normalized CBF values in Cluster 1 and Cluster 2 are shown in Figure 3 and Table 3. Compared to HC group (Cluster 1: 1.833 ± 0.141; Cluster 2: 1.823 ± 0.137), the normalized CBF values of Cluster 1 (1.493 ± 0.218) and Cluster 2 (1.542 ± 0.211) were both significantly decreased in SCD group with *P* < 0.0001.

**TABLE 3:** Results about ROI-based intergroup differences in CBF.

Regions	Normalized CBF		*T*-value	*p*-value
	SCD group	HC group		
Cluster 1	1.493 ± 0.218	1.833 ± 0.141	–7.179	<0.0001
Cluster 2	1.542 ± 0.211	1.823 ± 0.137	–6.127	<0.0001

### Correlations between cerebral blood flow and clinical characteristics

**FIGURE 4:**
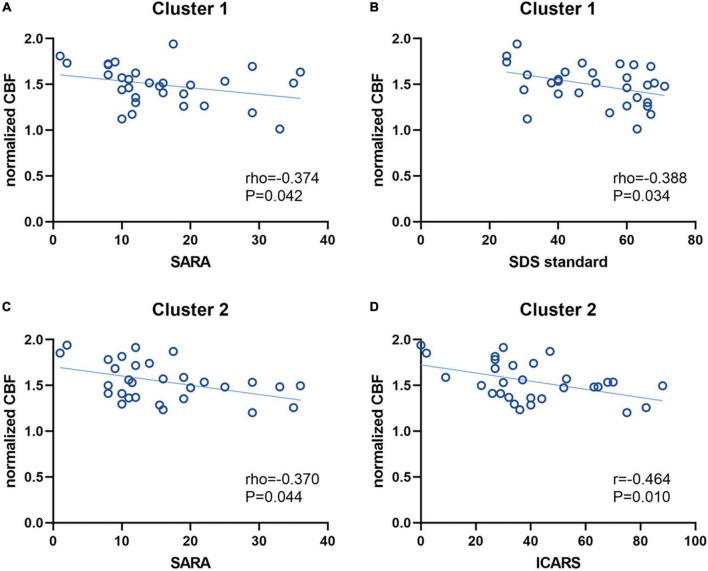
Correlations between the normalized CBF values and clinical characteristics. The normalized CBF of Cluster 1 had a negative correlation with SARA scores **(A)** and SDS standard scores **(B)**, respectively. For Cluster 2, the normalized CBF was also negatively correlated with SARA scores **(C)** and ICARS scores **(D)**, respectively. CBF, cerebral blood flow; SARA, scale for assessment and rating of ataxia; SDS, self-rating depression scale; ICARS, international cooperative ataxia rating scale.

The ROI-based correlations between normalized CBF values and the clinical-relevant demographic characteristics (disease duration), clinical severity (SARA and ICARS scores) or psychological scales (SDS, SAS and SRSS) were analyzed in both Cluster 1 and Cluster 2, with significant correlations depicted graphically in Figure 4. The normalized CBF value of SCD patients in Cluster 1 showed a negative correlation with SARA scores (Spearman’s rho = –0.374, *P* = 0.042; Figure 4A) and standard SDS scores (Spearman’s rho = –0.388, *P* = 0.034; Figure 4B), respectively. Although in Cluster 1 the normalized CBF was negatively correlated with ICARS scores (Pearson *r* = –0.226, *P* = 0.229), standard SAS scores (Pearson *r* = –0.245, *P* = 0.191) and SRSS scores (Pearson *r* = –0.149, *P* = 0.431), these correlations were not significant. For Cluster 2, the normalized CBF was significantly negatively correlated with both SARA scores (Spearman’s rho = –0.370, *P* = 0.044; Figure 4C) and ICARS scores (Pearson *r* = –0.464, *P* = 0.010; Figure 4D), without showing any correlation with those three psychological scales (standard SAS scores: Pearson *r* = –0.138, *P* = 0.467; standard SDS scores: Spearman’s rho = –0.174, *P* = 0.358; SRSS scores: Pearson *r* = –0.339, *P* = 0.067). For both two clusters, the negative correlations between normalized CBF and disease duration of SCD patients were statistically insignificant (Cluster 1: Spearman’s rho = –0.218, *P* = 0.248; Cluster 2: Spearman’s rho = –0.287, *P* = 0.124).

## Discussion

In this study, we detected a whole-brain pattern of CBF alterations and assessed the relationships between CBF and clinical characteristics in patients with SCD using the non-invasive PCASL imaging technique. Our main findings are that: (1) compared with healthy controls, SCD patients showed hypoperfusion mainly in two clusters, with the involvement of several cerebellar lobules, cerebellar nuclei (dentate and fastigial) and part of the midbrain; (2) decreased CBF was associated with increased disease severity in both cerebellar and midbrain clusters; (3) mental disorder in terms of depression status was correlated with CBF in the cerebellar cluster.

The whole-brain voxel-based analysis demonstrated decreased normalized CBF, which approximately reflected the decline of brain neuronal activity and was predominantly distributed in the cerebellar Cluster 1 (bilateral dentate and fastigial nuclei, bilateral cerebellar lobules I-IV, V and IX, left lobule VI, right lobule VIIIb and lobules IX and X of the vermis) and in the midbrain Cluster 2 (dorsal part of raphe nucleus). Although we did not detect any hemodynamic changes in the cerebral regions, the hypoperfusion areas in our study are consistent with neuropathological findings that neuronal cell losses of SCD often happened in the cerebellar cortex (Purkinje cells), spinal cord and brainstem nuclei but occasionally associated with degeneration of the basal ganglia and cerebral cortex (Yamada et al., 2016). In addition, our MR-based CBF decline in patients with SCD is also coinciding with previous reports using PET and SPECT (Sun et al., 1994; De Michele et al., 1998; Sidtis et al., 2010; Braga-Neto et al., 2012, 2016). A previous ROI-based retrospective ASL MRI study also showed significantly reduced mean normalized CBF in cerebellar cortices and crus in SCD (Ikawa et al., 2018). Furthermore, in patients with SCD subtypes, lower regional CBF (rCBF) values were detected by ASL in the ROIs of cerebellar cortex, cerebellar dentate nucleus, and pons in SCA3 (Xing et al., 2014) and by SPECT in the cerebellum and brainstem in MSA-C (Kimura et al., 2011). Compared to these studies that extracted and calculated CBF within their size-fixed or roughly hand-drawn single-slice ROIs, our data-driven voxel-level method could obtain the similar but more precise results and demonstrate the involved brain regions with more detail at the lobular level.

In cerebellar Cluster 1, affected cerebellar lobules I-IV and V belong to the anterior cerebellum which is thought to be important for motor control, whereas lobules VI, VIIIb and IX belong to the posterior cerebellum which is more involved in cognitive processing (Stoodley and Schmahmann, 2018). Moreover, the posterior cerebellum (lobules VIIIb, IX and X) was found to be the primary cerebellar targets of SCA 10 and the affected regions would expand to the anterior cerebellum in later stages (Hernandez-Castillo et al., 2019). Thus, hypoperfusion in these cerebellar regions may contribute to the clinical motor symptoms in SCD and its subtypes. Interestingly, after overlying our cerebellar results onto the cerebellar movement orientation flatmap (Diedrichsen and Zotow, 2015) generated by using task-based data from *N* = 100 participants scanned in the Human Connectome Project (Van Essen et al., 2013) to figure out the activation impairments related to the simple movements, we found a lot of overlap in the foot (bilateral), hand (bilateral, more severe in right) and tongue movement areas. The overlap indicates that the decreased CBF in these common regions may represent the drop of neuronal activity which could contribute to clinical motor symptoms in our SCD patients such as limb ataxias, dysarthria and dysphagia. Among those five simple movements, the involvement of bilateral feet seems most severe. And the non-symmetrically involvement of hand may result from the right handedness.

Apart from cerebellar lobules, the dentate and fastigial nuclei in Cluster 1 also demonstrated decreased CBF. The dentate nuclei (DN) of the cerebellum, which could project output to non-motor areas of the prefrontal and posterior parietal cortex (Bostan et al., 2013), are characteristic sites of neurodegeneration in several SCD subtypes such as SCA3 and Friedreich’s ataxia (Scherzed et al., 2012; Harding et al., 2016; Koeppen, 2018). The lower CBF in DN has also been detected using ASL in both onset and non-onset SCA3 patients (Xing et al., 2014). Together with DN, the fastigial nucleus (FN) is one of the ultimate integration stations and outputs of the spinocerebellum serving as a classical subcortical motor coordinator, holding a key position in the axial, proximal and ocular motor control by projecting to the medial descending systems and eye movement-related nuclei, and has also been implicated in the regulation of emotional activities (Zhang et al., 2016). Previous research on patients with SCA with saccadic intrusions (SCASI) also pointed out that FN played a critical role in eye movement, especially in programming sequences of saccades (King et al., 2011). It is worth noting that 86.7 percent of our SCD patients exist oculomotor deficits. And to the best of our knowledge, this is the first time that hypoperfusion has been identified in FN in patients with SCD. Thus, we speculate that the CBF decline in FN may contribute to motor symptoms especially the oculomotor deficits in SCD.

Similar to prior findings that identified decreased CBF in the brainstem (Sun et al., 1994; De Michele et al., 1998; Watanabe et al., 2005; Sidtis et al., 2010; Xing et al., 2014), our finding of Cluster 2 is situated in the midbrain of brainstem with the dorsal part of raphe nuclei involved in CBF decline. The raphe nuclei (RN), whose main neuronal components are serotonergic neurons, are distributed near the midline of the brainstem and the serotonergic projections participate in the regulation of motor, somatosensory and limbic systems and have been associated with sleep, depression and anxiety behavior (Hornung, 2003). Pathoanatomical studies suggested that marked neuronal loss in RN was observed in SCD, and damage to RN might contribute to the slowing of horizontal saccades (Rüb et al., 2003; Hellenbroich et al., 2006; Hoche et al., 2011). In addition, FN in Cluster 1 also receives serotonergic projections arising from the medullary/pontine reticular formation and RN (Bishop et al., 1988), which may indicates that hemodynamic damage of RN may affect the perfusion status in FN and the SCD-related eye movement function. According to a previous study, the baseline activity of raphe neurons increases in phase with swallowing (Ribeiro-do-Valle, 1997). And in this study, 46.7 percent of SCD patients suffered from dysphagia which may be associated with the decrease neuronal activity reflected by hypoperfusion in RN.

In terms of clinical characteristics, we found that SARA and ICARS scores were both significantly negatively correlated with the normalized CBF in Cluster 2. But in Cluster 1, only the SARA scores but not the ICARS scores significantly negatively correlated with the normalized CBF. The possible reason behind this might be the slight difference between ICARS and SARA. A previous study that compared ICARS scores with SARA scores among patients with SCD suggested that SARA was superior to ICARS in terms of practicability, reliability, and scale structure through assessing ataxia by the two scales at the same time, and might be more objective as the use of vague expressions reduced in SARA compared to that in ICARS (Yabe et al., 2008). Given that the significant relationship between increased SARA scores and decreased CBF in both Cluster 1 and Cluster 2, the MR-based CBF alteration detected by PCASL in the cerebellum and midbrain is likely to be the reflection of SCD disease severity. However, in this study, no significant correlations were found between disease duration and the normalized CBF (neither in Cluster 1 nor in Cluster 2), which is similar to another SCD study (Ikawa et al., 2018) where the correlation between disease duration and cerebellar CBF was insignificant as well no matter measured by ASL or SPECT. In addition, CBF of the cerebellum showed no correlation to the duration of the illness even if using PET in 23 patients with SCD (Sakai et al., 1989). One explanation for the insignificant relationship between decreased cerebellar/midbrain CBF and increased disease duration may be that the total number of SCD patients is relatively small (no more than 30 in those studies mentioned as well as ours), which may reduce the statistical efficiency. Another possible interpretation is that the progression speed of disease varies from subtype to subtype, which means patients who have the same disease duration might be in different disease stage. However, several studies regarded cerebellar CBF value as an imaging biomarker for the early stage of SCA3/SCD as the decline of CBF could appear in non-onset patients or even before apparent structural atrophy or disease onset (Sakai et al., 1989; Xing et al., 2014). Therefore, the present finding might indicate that the hypoperfusion in either cerebellum or midbrain would not continue to develop with the prolongation of SCD course.

As for non-motor psychological symptoms, a previous study summarized that 17–26 percent of SCA patients suffered from depression (Lo et al., 2016). Though depressive symptoms worsen with disease progression, the relationships between neurodegeneration, disease severity, motor disability and depression are complex (Schmitz-Hübsch et al., 2010; Klockgether et al., 2019). We further assessed the correlation between CBF and depressive status in this study. Although the affected lobules IX of the vermis of Cluster 1 was thought to be involved in emotional processing (Schraa-Tam et al., 2012) and interruption in brainstem raphe of Cluster 2 was highly prevalent in patients with depression associated with certain neurodegenerative diseases (Mijajlovic et al., 2014), SDS standard scores only significantly negatively correlated with the normalized CBF in the cerebellar Cluster 1 rather than the midbrain Cluster 2. Given that patients with depression displayed lower rCBF in cerebellum and thalamus in a PET investigation (Liotti et al., 2002), our finding may suggest the possible contribution of cerebellar hypoperfusion to emotional changes of depressive status in SCD. Except for depression, anxiety and impaired sleep quality are also present in vast majority of SCD patients (Pedroso et al., 2011; Silva et al., 2015; Yuan et al., 2019; Mastammanavar et al., 2020). However, no significant correlations were found between CBF and the rest psychological scales (SAS and SPSS) in either Cluster 1 or Cluster 2. Thus, whether hypoperfusion of cerebellum and midbrain is related to anxiety and sleep disorders still needs further verification. Overall, the decline of cerebellar CBF value in SCD patients could give clues to some mental health problems, and psychiatric interventions for those with lower CBF should be prioritized.

This study has a few limitations. First, the sample size of our study is relatively small, which might have reduced the statistical power in altered CBF assessment and correlation analyses. Further investigations in larger SCD cohort with subtype analyses are required to confirm the CBF changes and clinical correlates. Second, as a pilot study, we did not separate the gray and white matter during whole-brain analysis, and did not take the gray matter volume changes into consideration, further studies should analyze the cortical and subcortical CBF alterations with the control of the regional gray matter volume. Third, some of our patients received medications (such as coenzyme Q_10_, vitamin E and mecobalamin) to treat their ataxia symptoms, but the influence of drugs was often ignored in neuroimaging studies of patients with SCD because of the poor drug efficacy and the irregular medication. It is still not yet clear to what extent the finding and its interpretation can be influenced by medication, which need more rigorous research to explore. Finally, we only employed a single MRI modality to measure CBF with the cross-section study design to reveal the CBF alteration, follow-up longitudinal and multimodal studies might help in the future to differentiate perfusion signature of SCD from presymptomatic to late disease stages of the disease and monitor CBF changes with the disease progression.

## Conclusion

In conclusion, through the non-invasive ASL-MRI method, we investigate the altered whole-brain normalized CBF in SCD patients. We found decreased CBF in multiple cerebellar lobules and deep cerebellar nuclei as well as the midbrain of brainstem, and hypoperfusion in these regions was correlated with disease severity and depression scores, which may indicate deficits in motor and emotional conditions. MRI-based CBF value may be a promising neuroimaging biomarker to reflect the severity of neurodegeneration at the whole-brain level *in vivo*, and suggesting mental changes.

## Data availability statement

The original contributions presented in this study are included in the article/supplementary material, further inquiries can be directed to the corresponding author/s.

## Ethics statement

The studies involving human participants were reviewed and approved by the Ethics Committee of China-Japan Friendship Hospital. The patients/participants provided their written informed consent to participate in this study.

## Author contributions

BL and AY collected, processed, analyzed and interpreted data and images, wrote, and drafted the manuscript. WG and YC performed the MRI scanning and checked the image quality. YW, XL, and KL searched and managed the literature. LZ undertook neurological diagnosis and the acquisition of neuropsychology data. GM and LZ designed the study and critically revised the manuscript. All authors contributed to the article and have approved the final submitted manuscript.

## Footnotes

1
https://www.fil.ion.ucl.ac.uk/spm/software/

2
https://www.alivelearn.net/xjview

3
https://www.nitrc.org/projects/mricron/

4
https://www.diedrichsenlab.org/imaging/suit_flatmap.htm
